# Biographical Memoir of Mr. Wm. Tebb

**Published:** 1899-09

**Authors:** M. R. Leverson

**Affiliations:** Fort Hamilton, N. Y.


### BIOGRAPHICAL MEMOIR OF MR. WM. TEBB.

M. R. Leverson, M. D., Fort Hamilton, N. Y.

(Continued from July No., p. 316.)

In 1879, Mr. Tebb again visited this country for the purpose of trying to spread among us a knowledge of the real character of vaccination, as shown by the official statistics and blue-books.

Dr. Constantine Hering, of Philadelphia, Drs. Alexander Wilder, Robert Gunn, Jos. Dobson, Winterburn, Holhoohan, J. Emery Coderre, professor of "Materia Medica and Therapeutics," at the School of Medicine and Surgery at Montreal, under the impulsion given by Mr. Tebb, founded the Anti-Vaccination Society of America, with Dr. Wilder, professor of Physiology, for president.

After the departure of Mr. Tebb, the society languished until 1895, when it was reorganized with Dr. M. R. Leverson, of New York, as secretary and Dr. Barclay, of Newburg, president. This gentleman, unfortunately, thought his duty complete when the anti-vaccinators of Newburg triumphed in the local election, and secured a school board pledged to disregard the law requiring the vaccination of school children.

Mr. Piehn, of Nora Springs, Iowa, is at present the very active president of the Society, Dr. Leverson and Mr. L. J. Weyprecht, of New York, and Dr. Clausen, of St. Ansgar, Iowa, act as vice-presidents. Frank D. Blue, of Terre Haute, Indiana, is indefatigable as secretary, and Mr. E. C. Townsend, of New York, formerly editor and proprietor of the *Anti-Vaccination News*, is assistant secretary for the Eastern States.

International Anti-Vaccination Congresses were held in Cologne, Berne, Charleroi, and Paris under the presidency of Dr. Hubert Boëns, and the reports of the proceedings were sent to leading government officials, and the chiefs of Public Health Departments in all countries.

Dr. Boëns was at the time of his death the oldest veteran among the numerous men of science who have opposed vaccination. Herbert Spencer and A. R. Wallace, declared themselves in opposition thereto later in point of time to Dr. Boëns, who was also the first, not only to detect the resemblance amounting to an almost identity between cow-pox and syphilis, but also to trace the frequent origin of so-called spontaneous cow-pox to actual syphilis in man.

The Sixth International Anti-Vaccination Congress was held in Berlin, in September, 1899. It had been announced for June, 1899, and delegates from the United States had actually started to attend it when they were informed of its postponement until September. As a consequence, the United States were not represented at the conference, though Dr. Leverson forwarded a paper which he had intended to read had he been present.

The agitation in England was actively continued, and after many years of struggle, the people succeeded in electing guardians pledged not to enforce the compulsory law in Banbury, Gloucester, Leicester, Keighley, Oldham, and many other places; and in Leicester in 1885, a public demonstration was held in which 50,000 of the inhabitants took part, with delegates from all parts of the United Kingdom. The procession was over a mile long, and displayed hundreds of flags and banners with pictorial allusions in every form of serious and grotesque attack upon the Jennerian system. Among these were the following: "Sanitation not Vaccination," "Better a Felon's Cell Than a Poisoned Babe," "It Is Not Small-pox You Are Stamping Out, But Human Creatures' Lives," "Revolt Against Bad Laws Is a Christian Virtue and a National Duty." The great procession assembled in the market-place of this ancient city, when speeches were made by the leaders of the movement, and the vaccination acts were publicly burned amidst the cheers of the vast population assembled, and in the presence of the mayor and other public officials. This was the greatest public demonstration that has ever been held in the history of this midland town.

The result of the demonstration, which was fully reported in *The Times* and in every leading paper in the country, was that the Leicester Board of Guardians, whose office is to enforce the vaccination law, refused to prosecute the conscientious and intelligent objectors, and the law became a dead letter. From that time forth, it was found impossible for the authorities not only in Leicester, but in many other towns, to enforce vaccination upon unwilling parents, many of whom had become opponents of the Jennerian rite, by having had their children injured or killed by vaccination. The troubles of Leicester, we regret to note, are not yet over.

In July, 1898, an amendment to the vaccination acts was proposed in Parliament by Mr. Pickersgill, prohibiting all prosecutions for neglecting to vaccinate a child except upon permission for such prosecution given by the guardians, *i. e.*, the local authority. Mr. Chaplin, the president of the Local Government Board, gave a pledge, or what at least was understood as a pledge, by members of all parties in the House that so long as he held office there should be no attempt to enforce vaccination, or for the vaccinating officer to prosecute without the consent of the guardians. This construction was put upon his words by several members in Mr. Chaplin's presence, in appealing to Mr. Pickersgill to withdraw his amendment as being unnecessary in the face of such a pledge. Mr. Chaplin heard these remarks and made no sign. Hansard reported Mr. Chaplin's speech in the same sense, and there appears the asterisk used by the publishers to denote that the speaker had revised the proof. In the teeth of this, the Local Government Board now calls on the vaccinating officers to prosecute without any authority from the guardians, and Mr. Chaplin says that Hansard's report and the testimony of numerous members of both sides of the House are inaccurate. In this state of things the vaccination officer of Leicester resigned, and the guardians have declined to appoint a successor except upon the condition that he do not prosecute except as authorized by them. To this the Local Government Board have refused assent and have mandamused the Board to appoint a vaccination officer. The mandamus has been made absolute. What course the guardians will now take is not yet known. One thing, however, is as certain as anything human can be, and that is that the noble men and women of whom the Leicester Board of Guardians is composed will one and all go to prison before they will appoint a legal poisoner of the children of their city.

The case is an instructive one from several other points of view. 1st. As emphasizing the importance of local self-government; 2d. As demonstrating the absurdity of the machinery of government adopted by a race which arrogates to itself special and almost exclusive knowledge of self-government; 3d. The ignorance of the law-makers of England of the science of legislation. But this ignorance, almost universal as it is in England, is still more so in the United States,—perhaps we might add arrogance as well with regard to the very subject upon which their ignorance is most complete!

Thus, before the Royal Commission was appointed, the vaccination laws had become inoperative in about one hundred and twenty towns and districts in England, and the Government had no alternative except to appoint a Commission of Inquiry, which lasted seven years, and is the first and only exhaustive investigation made in any country as to the results of vaccination. Lord Herschell, formerly Lord Chancellor of England, was the chairman of the Commission. Upon this Commission only a single representative of the antivaccination movement was given a seat, while twelve at least of its fifteen members were known believers in the efficacy of vaccination as an antidote to small-pox.

More than one hundred witnesses, including medical men of high position, gave evidence to show that vaccination neither mitigated nor prevented small-pox, but that it was instrumental in spreading inoculable diseases, which diseases had seriously increased, *according to official statistics*, from the year vaccination had been made compulsory. The most important recommendation of the Commission was that a law should be passed allowing conscientious objectors to make a declaration before a magistrate, and thus escape prosecution and judicial penalties.

The discussion in both houses of Parliament on this question was considered to be the most interesting and important of the session. Mr. Balfour, the leader of the House of Commons, and Lord Salisbury, the Prime Minister, and leading members of both the Government and Opposition benches, spoke strongly in favor of this recommendation, and, on the 12th of August, last, the Queen appended her signature to the bill, and thus put an end to some of the worst features of vaccination tryanny in England. Vaccination, however, is still in force in the army, navy, and civil service, and is also obligatory upon pupil-teachers in the Board Schools, but Mr. Tebb is of the opinion that this anomaly will not long be permitted to survive.

During the numerous conferences organized by the anti-vaccination party in London, medical men and persons interested in the public movement were invited to give their views. On several occasions visitors from the United States attended and furnished details of the oppression and injustice which many persons have to undergo in various States of America, by reason of the public schools being closed to the children of parents who object to vaccination. These parents stated at the conference that they were obliged to send their children to private schools at considerable expense, and in the case of poor parents they said the hardships were more acute, as the children must either be submitted to an ordinance to which they conscientiously objected or consigned to ignorance. There were even stronger features of the vaccination tyranny disclosed by some of the speakers, who called attention to the fact that in some States fines were imposed on those who refused to submit to vaccination, and that vaccination has on numerous occasions been forced *vi et armis* with the aid of the police. The fines are cumulative and crushing in their severity.

The subject of vaccinating immigrants before permitting them to land in the United States has frequently been brought to notice in the English and Continental press, and before the International Anti-Vaccination Conferences, which have been held in various parts of Europe. At the Congress held in Berne, Switzerland, in 1883, a deputation of medical men, appointed by the Congress, waited upon the American Minister and called his attention to numerous cases of injury, some incurable, induced by vaccination as enforced at the Atlantic ports of the United States. A memorial was afterward drawn up at the request of the Minister, giving details of these cases of injury, with photographs of the suffering victims, for the purpose of presenting them to the Health Department at Washington. Since then various efforts in the same direction have been made, but unfortunately without result.

Mr. Tebb has recently paid another visit to the United States, accompanied by his wife, in furtherance of the emancipation of the people from the gross superstition forced upon them. He arrived in Boston on the steamship "Canada," September 16th, 1898. On the previous day, all the immigrant passengers, about five hundred in number, were examined and those not showing recent vaccination marks were compulsorily vaccinated, in spite of strong expressions of indignation at the injustice to which they had to submit; the saloon passengers, meantime, many of whom were as much opposed to vaccination as is Mr. Tebb, were none of them interfered with. This discrimination shows that in respect to vaccination, there is one law for the rich and another for the poor. It may be remarked here that in no other country in the world do immigrants have to submit to a like ordeal.

Mr. Tebb is of the opinion that if a summary of the facts that were laid before the Royal Commission could be reprinted in America and studied by the reflective portions of our citizens, vaccination would be abandoned to as great an extent as it has been in England, and that the people would be liberated from what many regard as an odious and indefensible tyranny.

An abstract of all this testimony is now in course of publication in The Homœopathic Physician, but unhappily, the general apathy of the people of America to what concerns the general good and even their own rights and duties as parents as well as to the well-being of their children, presents an obstacle which it will be hard to overcome.

While essentially a *people's question* and one which, as a subject for legislation, the common people are as competent to judge of as is the most learned physician, and in many cases more so, it is well to bear in mind that the opposition to vaccination numbers within its ranks many of the most distinguished men in medicine and science. Among them may be reckoned the late Drs. Hamernick, of Prague; Ancelon, of Nancy, France; Hubert Boëns, of Brussels; Professor Vogt, of Berne; Professor Emery Coderre, M. D.; Dr. Aitkin; Dr. Charles Creighton, formerly demonstrator in anatomy at Cambridge University and a prominent writer on pathology in the *Encyclopædia Britannica;* Professor Crookshank, the eminent bacteriologist of King's College, London; Dr. Alfred R. Wallace, F. R. S., the co-discoverer with Darwin of the doctrine of evolution; Dr. Garth Wilkinson, described by Emerson, in his volume entitled *English Traits*, as the greatest writer of English since Bacon; Professor Francis W. Newman; John Stuart Mill, and Herbert Spencer, all of whom have been opposed not only to compulsory vaccination, but have had no faith in vaccination as a preventative of small-pox. The Right Honorable William E. Gladstone believed that the value of vaccination has not been sufficiently proved to warrant making it compulsory, and he distrusted the practice.

But the plain truth of the matter is, *that almost every man of science, who has carefully investigated vaccination at any time within the past forty years, has condemned the practice.* Yet, in the teeth of this fact, the official doctors refuse to investigate, and the majority of the daily press refuses to publish anything which tells against this unphysiological rite, worthy only of an uncivilized tribe of savages.

The medical officers in the vaccination department of England hoped to get rid of the objection to vaccination by introducing a new system of vaccine lymph made in Germany; namely, vaccine matter mixed with glycerine. But, in Mr. Tebb's evidence before the Royal Commission, he produced an official report, issued by the Board of Health of Berlin, of two hundred and forty cases of injury at the Isle of Rügen, North Germany, due to this variety of poison, made or produced from a Government establishment at Stettin, North Germany, and in a volume recently published, entitled, *A Century of Vaccination*, Dr. W. Scott Tebb, M. A., of Cambridge University, shows conclusively by abundant evidence that this variety of vaccine has no advantage in point of safety over the now discredited arm-to-arm virus. Small-pox and other zymotic diseases are not prevented by the inoculative method, nor by vaccine or any other virus, but by personal and municipal sanitation. Epidemics occur from foul air, defective house drainage, insufficient and improper water, and overcrowding. These outbreaks are prevented by the introduction of public baths and wash-houses, and the increase of open spaces and parks, scientific sewerage, and the removal of rookeries, and to these remedies public attention is now being extensively directed by municipal authorities all over the United Kingdom. It is estimated that £300,000,000 have already been expended in this important work in the last thirty years in England alone, with the result that the death-rate has enormously diminished, and it must be noticed that the death-rate in Leicester, the centre of the anti-vaccination agitation, which when nearly all the people were vaccinated, and when vaccination was considered a preventative of small-pox, was over twenty-six per thousand, has fallen to fifteen per thousand, with a downward tendency, since its abandonment.

Drs. Close, Foote, Dobson, Leverson, and others of the leading opponents of vaccination in New York, were desirous of tendering a public reception to Mr. Tebb, but the state of his health rendered it impossible. Though constantly suffering, he visited several of the chief cities of the East, as well as Washington, D.C., and Richmond, Va., Baltimore, and Philadelphia, and was interviewed by reporters in nearly all those cities, though frequently racked with pain while submitting to the interviews. He paid few visits while in the United States, but among the few he called on was the widow of his friend, the friend of man, Henry George, at her home at Fort Hamilton.

In the various interviews which Mr. Tebb gave to the representatives of the press Mr. Tebb modestly disclaimed much of the merit he really deserves for the progress and success of the agitation against compulsory vaccination in England.

He entered upon the work nearly thirty years ago, upon the instigation of his wife, after it had been well started by the Gibbses, the Hume-Rotherys, the Dornbrusches, Mr. Alex. Wheeler, the Countess de Noailles, and other pioneers, whose work and worth are recorded by William White in his classical work, *The Story of a Great Delusion*, which should be read by every one who cares to understand how so monstrous a delusion as that of intentional blood poisoning came to prevail. To the student of the human intellect also, a study of that work is invaluable, as showing how "myths" can arise in semi-scientific times and be maintained in what is called a scientific age in the teeth of facts and of reason.

Mr. Tebb considers that he has only been the mouthpiece of his defenceless and persecuted fellow-countrymen, who by their sufferings and devotion have borne the brunt of the fight during a long and arduous struggle. But in truth he has been much more than all that. He was himself one of the early martyrs to the tyrannical law which was surreptitiously smuggled through the British legislature, the passage whereof illustrates some of the defects of British and American procedure against which the wise provisions of the great legist Bentham proffered an altogether neglected protection.

Mr. Tebb, in his investigations into the revival of leprosy, has also done much original work which but for him would have been left undone.

Accompanied by his wife, Mr. Tebb left New York for Southampton on the "St. Louis" on the 16th of November, and reached his home, Rede Hall, Surrey, England, upon the 23d of November, arriving in somewhat better health. There we leave him, wishing, not more for his own sake than for that of humanity, that he may yet be spared many years in restored health, to continue the part he has so manfully played during the past fifty years for the well-being and advancement of mankind.

The union of Mr. and Mrs. Tebb has been blessed with four children, three daughters and one son. His son, Dr. W. Scott Tebb, became a physician, having graduated from Cambridge University, where the reigning superstition continues to be taught as an incontrovertible dogma. He emancipated himself from its chains, and in a volume of 450 pages, entitled, *A*

*Century of Vaccination*, has further pulverized beneath the weight of statistical science and logic this hydra of vaccination. Conceived in ignorance, delivered in fraud, sustained by falsehood, greed, and physical force, it will be forever the scandal and opprobrium of the nineteenth century, and of the people who have submitted to so shameful and tyrannical a yoke.

---

LIQUEFIED AIR.—The process of liquefying atmospheric air has by a recent invention become so simplified that it can now be produced in great adundance and at a comparatively trifling cost. We understand a plant is now being erected in Los Angeles with a capacity of 1,500 gallons a day. The uses to which this agent can be applied as a force easily directed and controlled will be increased with study and experience. Already our physicians are testing its power, with the most satisfactory results, as a cooling agent in the sick room, as a clean caustic and as a destroyer of microbes. Dr. Murray, of the City Hospital, recently obtained excellent results from the application of liquid air to several cases of migratory erysipelas. It was lightly brushed over the surface and seemed to speedly control the inflammation. Equally good results were obtained in sloughing ulcers which had proved intractable to other treatment. Dr. Murray thinks the intense cold freezes the bacteria without producing appreciable harm to the living tissue, and suggests that in this powerful agent we may possibly find a controlling power in the treatment of leprosy, a disease which has thus far baffled the world. It is very evident that this new agent is yet to play a most important part, not only as a mechanical force, but in the treatment of diseases.—*Medical Times*, *Sept.*, *1899*.

